# Immunomodulation by Human Milk Oligosaccharides: The Potential Role in Prevention of Allergic Diseases

**DOI:** 10.3389/fimmu.2020.00801

**Published:** 2020-05-07

**Authors:** Marit Zuurveld, Nikita P. van Witzenburg, Johan Garssen, Gert Folkerts, Bernd Stahl, Belinda van't Land, Linette E. M. Willemsen

**Affiliations:** ^1^Division of Pharmacology, Faculty of Science, Utrecht Institute for Pharmaceutical Sciences, Utrecht University, Utrecht, Netherlands; ^2^Global Centre of Excellence Immunology, Danone Nutricia Research B.V., Utrecht, Netherlands; ^3^Global Centre of Excellence Human Milk Research and Analytical Sciences, Danone Nutricia Research B.V., Utrecht, Netherlands; ^4^Division of Chemical Biology and Drug Discovery, Faculty of Science, Utrecht Institute for Pharmaceutical Sciences, Utrecht University, Utrecht, Netherlands; ^5^Center for Translational Immunology, University Medical Center Utrecht, Utrecht, Netherlands

**Keywords:** human milk oligosaccharides, mucosal immunity, allergic diseases, early life nutrition, sialyllactose, fucosyllactose, non-digestible oligosaccharides

## Abstract

The prevalence and incidence of allergic diseases is rising and these diseases have become the most common chronic diseases during childhood in Westernized countries. Early life forms a critical window predisposing for health or disease. Therefore, this can also be a window of opportunity for allergy prevention. Postnatally the gut needs to mature, and the microbiome is built which further drives the training of infant's immune system. Immunomodulatory components in breastmilk protect the infant in this crucial period by; providing nutrients that contain substrates for the microbiome, supporting intestinal barrier function, protecting against pathogenic infections, enhancing immune development and facilitating immune tolerance. The presence of a diverse human milk oligosaccharide (HMOS) mixture, containing several types of functional groups, points to engagement in several mechanisms related to immune and microbiome maturation in the infant's gastrointestinal tract. In recent years, several pathways impacted by HMOS have been elucidated, including their capacity to; fortify the microbiome composition, enhance production of short chain fatty acids, bind directly to pathogens and interact directly with the intestinal epithelium and immune cells. The exact mechanisms underlying the immune protective effects have not been fully elucidated yet. We hypothesize that HMOS may be involved in and can be utilized to provide protection from developing allergic diseases at a young age. In this review, we highlight several pathways involved in the immunomodulatory effects of HMOS and the potential role in prevention of allergic diseases. Recent studies have proposed possible mechanisms through which HMOS may contribute, either directly or indirectly, via microbiome modification, to induce oral tolerance. Future research should focus on the identification of specific pathways by which individual HMOS structures exert protective actions and thereby contribute to the capacity of the authentic HMOS mixture in early life allergy prevention.

## Introduction

Human milk is unique in its composition as it covers all nutritional and physiological infant requirements during the first months of life (1). Therefore, investigating the biological activity of components derived from human breast milk is an area of great interest, in order to identify specific components that support proper immune development in the infant when breastfeeding is not possible. The first indications of a link between breastfeeding and allergy outcome later in life has been published almost a century ago (2). Since then, numerous studies have been conducted to substantiate this suspected link (3–8). Breastmilk is the gold standard in early life nutrition, because of its large range of bio-active protective nutrients essential for healthy development of the microbiome and gastro-intestinal and immune maturation. However, it can also transfer allergens which may cause allergic reactions in atopic or allergic infants. Therefore, the conflicting data presented by these studies demonstrate the importance of studies further evaluating the biological activities of specific constituents found in human milk (9), such as human milk oligosaccharides (HMOS).

HMOS are the third most abundant component of human breast milk after lactose and lipids. The concentration of total HMOS in human breast milk ranges from 5 to 15 g/L, depending on the stage of lactation and genetic background of the mother (10, 11). More than two hundred structurally different forms of HMOS have been identified (12–14). Different structural and functional groups of HMOS have been related to various effects on several aspects of the immune system (15–19), highlighting the need for a diverse mixture of oligosaccharides in neonatal nutrition for optimal immune development.

Maturation of the immune system in the gastrointestinal tract is linked to proper systemic immunity and the establishment of effective oral tolerance for harmless food proteins and commensal bacteria of the host microbiome (20). As microbial colonization coincides with a rapidly maturing immune system in infants, microbial dysbiosis may therefore disturb development of the gastro-intestinal tract and immune system (21). Microbial dysbiosis and immature immune responses are thought to play a crucial role in e.g., necrotic enterocolitis (NEC), a disease characterized by inflammation and necrosis of the intestines affecting especially premature infants (22), whose immune system is not yet fully developed. Pathologies such as NEC and allergic diseases share common ground, as both have been linked to impaired microbial colonization and improper immune maturation.

One of the specific contributions of HMOS in human milk is its prebiotic capacity. Modulation of the infant's microbiome composition into a bifidogenic profile has been shown to have beneficial effects on infant health. Therefore, prebiotics, such as galacto-oligosaccharides (GOS) and fructo-oligosaccharides (FOS), have shown several beneficial immune and microbiome developments in infants (23–25). The specific combination of 90% short-chain (sc)GOS with 10% long-chain (lc)FOS resemble the molecular size distribution of the neutral HMOS fraction found in human milk (26). Prebiotic supplementation with scGOS and lcFOS reduces the incidence of allergy development (26–31). Murine models for both food allergy and house dust mite induced allergic asthma demonstrated the preventive effects of non-digestible oligosaccharides (29, 30). Moreover, scGOS/lcFOS supplemented infant formula in neonates decreased the prevalence of atopic dermatitis and other allergic manifestations (26–28).

Currently, only a small number of *in vivo* studies have investigated immunomodulatory properties and immune development capacities of HMOS. Thus, there are a limited amount of studies that attribute immune development properties to HMOS and individual HMOS structures. Several studies describing immunomodulatory effects of scGOS and lcFOS have been included in this review as they may serve as a framework in which future research could focus on elucidating how immune related mechanisms may be affected by HMOS. In addition, almost no clinical trials have investigated the effects of HMOS supplementation, although the association between the presence of specific HMOS biologically available in human milk and the prevalence of infectious diseases (32–34) or allergic diseases (35–37) has been indicated. The possible biological functions of HMOS gain support from studies that show a potential protective effect of prebiotic administration in *in vitro* models, animal models and human studies against development of asthma or allergy (28, 35, 38, 39). Most of the HMOS are not digested in the upper part of the gastrointestinal tract, but are fermented by local microbiota (40). A large proportion of HMOS will reach the colon intact (40), where they can serve as prebiotics for the colonic microbiota of the infant. Although a large portion of HMOS is metabolized by gut microbiota, some cross the intestinal (sub)mucosa and enter systemic circulation (13, 41, 42), thereby potentially modulating systemic immune functions. This means that HMOS may influence immunity and potentially not only the intestinal microbiome but also the microbiome composition in the lungs, providing a possible explanation for the observation that breastfed infants are less likely to develop asthma during childhood (43). In addition, reduced occurrence (up to 50% reduction) of atopic dermatitis, asthma, recurrent wheeze and food allergy in infants supplemented with prebiotics in early life has been observed (27, 28, 44–46). Despite these observations, little is known regarding the systemic distribution of HMOS in the infant, and how it may influence processes outside the gastrointestinal tract.

**Figure 1:**
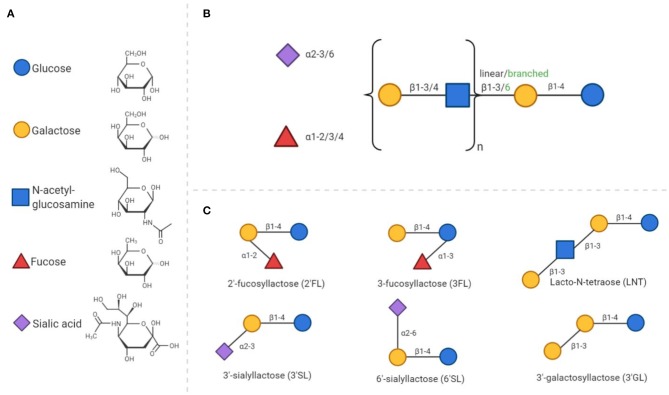
General composition of human milk oligosaccharides and synthetic analogs. **(A)** All HMOS consist of only 5 different monosaccharides. The chemical structures of these monosaccharides are presented in a D- configuration. **(B)** The composition of HMOS follows a distinct structure. Elongation of the core structure and decoration with fucose and/or sialic acid residues leads to the large number of different structures discovered to date. **(C)** As examples, six simple oligosaccharide structures are displayed.

The complexity and abundance of oligosaccharides in human milk is unique amongst mammals (47). HMOS play an essential role in the postnatal growth and development of the mucosal immune system. HMOS are made up of monosaccharide units such as glucose (Glc), galactose (Gal), fucose (Fuc), *N*-acetylglucosamine (GlcNAc), and sialic acid with *N*-acetylneuramic acid (Neu5Ac). HMOS synthesis follows a distinct pattern of formation. Each structure has a Gal-Glc unit at the reducing terminus, also known as a lactose unit, containing a β1–4 glycosidic linkage. Elongation of lactose can occur by addition of Gal-GlcNAc units via a β1–3 or β1–6 glycosidic bond to form the linear or branched core structures (see Figure 1). The HMOS core structure can be further modified through the addition of Fuc or Neu5Ac residues (48).

The unique diversity of HMOS also includes galactosyllactoses, with structures based on the elongation of lactose and further galactose residues (49, 50). These types of linkages are indigestible, but fermentable by specific bacteria; leading to the large number of ~200 distinct structures identified to date. Decoration of the core structure with sialic acid, results in an acidic structure, whereas all other HMOS, including those containing fucose groups, are considered neutral. The composition of HMOS produced by a mother is determined by genetic polymorphisms in genes encoding fucosyltransferases FUT2 [Secretor (Se) gene] and FUT3 [Lewis (Le) gene]. Both genes are polymorphic, the individual expression of these genes are accountable for variable enzyme activity and corresponding variation in HMOS profiles in breast milk (11). Recent data has even indicated that these genetic polymorphisms in mothers, impact immunologic outcome of their children later in life. This effect was demonstrated in children, with a hereditary high risk of developing allergic diseases, who were fed breast milk of FUT2 expressing mothers which decreased the incidence of allergic manifestation of these children at 2 years of age (36). However, from this study it cannot be concluded that solely this genetic polymorphism is related to the allergic outcome of the infant, as many genetic, nutritional and environmental factors contribute to the immune development in neonates.

Synthetically manufactured HMOS or HMOS produced by genetically engineered bacteria, such as 2'-fucosyllactose (2'FL) (51), 3-fucosyllactose (3FL) (52, 53), lacto-N-neotetraose (LNnT) (54), 3'-sialyllactose (3'SL), 6'-sialyllactose (6'SL) (55), and 3'-galactosyllactose (3'GL) (56) have become commercial available just recently. This provides the opportunity to study specific pathways by which individual HMOS structures exert their protective immunologic effects in infants.

## Allergic Sensitization and the Role of the Epithelial Barrier

The prevalence of allergic diseases is rising tremendously, particularly in Westernized regions (57). An allergic disease is an immunological result of complex interactions between genetic, environmental and lifestyle factors mainly triggered by harmless substances (58). Reduced microbial exposure and diversity is one of the many factors that may contribute to the rise in allergic disease prevalence. In allergic sensitization, a harmless, for example food-derived or airborne protein, crosses the mucosal lining and is presented by antigen presenting cells that drive T helper 2 (T_H_2) biased immunity contributing to IgE isotype switching of B-cells. Mucosal surfaces with epithelial barriers provide the body with protection from external factors, ensuring that only specific components and nutrients can pass through the epithelium and enter systemic circulation. Allergic sensitization has been linked to dysfunction of the epithelial barrier, both in the intestine and skin (59, 60). Epithelial barrier integrity depends, among other factors, on the mucus layer covering the single layer of epithelial cells. The mucus layer in the intestines prevents the majority of pathogens and intestinal contents from making direct contact with the epithelial cells (61). In humans, the most abundant protein present in the intestinal mucus layer is mucin 2, which is secreted by goblet cells (62). Several factors, including the microbiota, can influence the composition and therefore the protective effects of the mucus (63). Gut maturation takes place the first couple of weeks after birth rendering a leaky barrier in the first weeks of life (64). This can help to organize oral tolerance induction, but it also provides a risk for allergic sensitization.

Tight junctions strengthen apical connections between epithelial cells that cover the underlying connective tissue, thereby contributing to barrier function. Epithelial tight junction proteins tightly regulate paracellular compartments, preventing transport of large molecules, such as proteins and lipids or microbes and microbial products into the underlying tissue (65). These tight junctions are apically present and are crucial for epithelial barrier integrity. Upon epithelial injury, antigens can cross the epithelium more easily. Cytokines, such as interleukin-8 (IL-8), IL-25, IL-33, and thymic stromal lymphopoietin (TSLP), are produced by the epithelial cells as a response to stress and damage (66). These epithelial cell secreted cytokines influence neighboring dendritic cells (DCs) (67). Generally, DCs in the gastrointestinal tract are hyporesponsive and favor tolerogenic response to prevent unnecessary inflammatory responses to antigens and microbes (68). IL-25, IL-33, and TSLP stimulate the uptake and processing of foreign antigens by DCs and drive these DCs to promote development of T_H_2 cells from naïve T cells (69, 70). Consequently, IL-4 and IL-13 produced by the T_H_2 cells induces the activation and class-switching of B cells to produce allergen-specific IgE (67). The secreted IgE will bind to the high-affinity Fc receptors on the surface of mast cells. Upon a consequent encounter, the allergen crosslinks the IgE bound to the mast cells, triggering the mast cell to degranulate and release inflammatory mediators, such as histamine, causing the symptoms of allergic disease (71).

Newborns may be particularly susceptible to developing allergic diseases since the immune system after birth is dominated by T_H_2 responsiveness (72). Immune maturation involves shifting toward a more T helper 1 (T_H_1) prone and regulatory type, which favors the development of adequate immune protection and balanced immune responses (73). The importance of the epithelial barrier and mucosal homeostasis in prevention of allergic sensitization has sparked interest. HMOS may help to support this function by stimulating proper epithelial maturation and microbial colonization (74–76).

## HMOS Shape the Microbiota of Neonates

The first 1,000 days of life are critical for the development of a diverse, stable gut microbiome (76–78). The initial microbial composition of the gut is determined by host genetics and environmental factors, such as health status, mode of delivery and diet (79). The first bacteria to colonize neonate's intestines are *Enterobacteriaceae* and *Staphylococcus* (80), followed by bifidobacteria and lactic acid bacteria (81). Proper colonization is essential for optimal development and health, as the establishment of a rich and diverse microbiome is related to a decreased prevalence of allergic (82), metabolic and other immunologic diseases later in life (83, 84).

**Figure 2:**
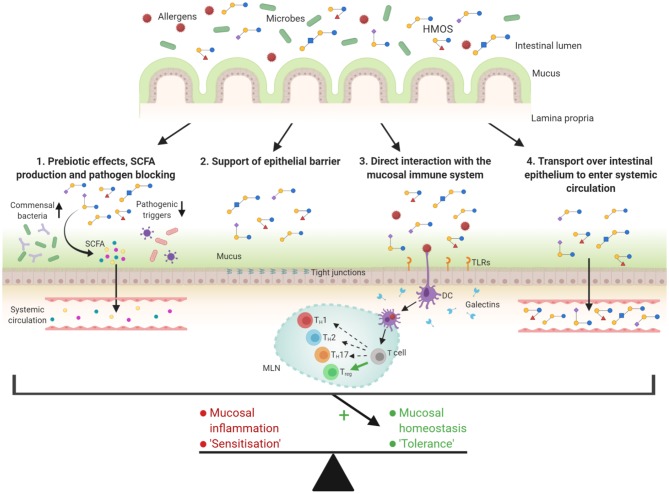
Overview of the possible functions of HMOS related to the prevention of allergic diseases. The diversity in structures suggests engagement in several mechanisms related to maturation of the infant's gastrointestinal tract. (1) HMOS have shown to function as prebiotics and therefore stimulate growth of commensal bacteria. In addition, HMOS have shown to bind pathogens, thereby preventing binding of these pathogens to the intestinal epithelium itself and possible consequent infections. SCFAs produced during HMOS fermentation can enhance epithelial barrier integrity and locally and systemically modify immune responses. (2) HMOS can promote mucus production and epithelial tight junction integrity, thereby supporting the physical barrier between the intestinal epithelium and the gut content. (3) Several mechanisms by which HMOS directly affect the immune function have been described. Modulation of the response of DCs is one of those described mechanisms which may be relevant for the instruction of protective mucosal immune development. (4) Transportation of a small fraction of HMOS over the intestinal epithelium, results in systemic availability of these structures. This suggests an immunomodulatory role for HMOS, also beyond the gastrointestinal tract. All these HMOS related mechanisms can potentially enhance tolerance induction and therefore possibly prevent allergic diseases. Adjusted from Ayechu-Muruzabal et al. (48).

HMOS promote the growth of beneficial bacteria, such as *Bifidobacterium* and *Lactobacillus* species (85, 86). Therefore, HMOS are known for their prebiotic effects and as players in shaping the microbiota of infants as depicted in Figure 2. The microbiota supporting effects of HMOS were observed when the gut colonization in breast-fed and formula-fed infants was compared, while addition of scGOS/lcFOS to formula milk was found to bring the microbiome composition closer to that of breastfed infants (87, 88). The microbiota are capable of fermenting oligosaccharides, however the capacity to degrade HMOS is strain-specific and depends on the presence of several genes (89, 90). Several strains of *Bifidobacterium* are well-adapted to digest purified natural HMOS into metabolites such as short chain fatty acids (SCFA) (90–93). Glycosyl hydrolases (GH), expressed by bifidobacteria, cleave monosaccharides from the HMOS and making them available for utilization by the microbe (94). This enzymatic degradation can either occur by membrane-associated extracellular GHs (95) or, as is the case for *Bifidobacterium infantis*, intact HMOS are transported into the cell by Solute Binding Proteins (96) and broken down by GHs inside the cytoplasm (97). The available monosaccharides are assimilated in central metabolic pathways and consequently release large volumes of e.g., SCFAs (98).

Both *B. longum* and *B. bifidum*, the major intestinal bacteria found in breastfed infants, are remarkably well-equipped to metabolize HMOS. In contrast, *B. adolescentis* is often associated with the adult intestinal microbiota, and is a less effective HMOS metabolizer (81, 91, 93). In contrast to *Bifidobacterium* spp., *Bacteroides* spp. are not specifically adapted to metabolize HMOS, but degradation of plant polysaccharides by *Bacteroides spp*. has been indicated (90). As plant-derived oligosaccharides are structurally comparable to human oligosaccharides, the capacity of multiple *Bacteroides* strains to metabolize HMOS is not unexpected (89). Providing a substrate for commensal gut bacteria results in a competitive growth advantage for these bacteria, enhancing proper colonization in the infants intestine and reducing growth conditions for and colonization by pathogenic bacteria (99, 100).

Unlike several species of commensal gut bacteria discussed previously, certain pathogenic species do not use HMOS as carbohydrate source for growth, including *Clostridium difficile, Enterococcus faecalis* and *Escherichia coli* (89). In addition, HMOS can actively bind to several pathogenic microbes and thereby possibly prevent adhesion as first step of infection (101). Infant formula can be supplemented with the prebiotics scGOS and lcFOS in order to promote the growth of various *Bifidobacterium* and *Lactobacillus* strains (102). However, these oligosaccharides do not contain terminal fucose or sialic acid residues, hence missing out biological function of HMOS related to these specific functional groups (103).

Proper colonization of the gut promotes intestinal barrier function and immune maturation (104). The establishment of a rich and diverse microbiome is related to a decreased prevalence of allergic diseases (82). Prebiotics like HMOS can support the growth and function of commensal bacteria and therefore possibly enhance gut microbial diversity. The association between microbial diversity and development of allergic diseases (83, 105) and the role of HMOS in this context, has yet to be elucidated.

## Metabolites of HMOS Influence Intestinal Barrier Integrity and Immune Function

As described in previous section, HMOS are digested by intestinal bacteria, resulting in various metabolites, among which SCFA are well-known for immunomodulatory properties. The fermentation of major HMOS by bifidobacteria and lactobacilli into SCFA is very efficient (81), hence these bacteria are the dominant suppliers of SCFA in the infant's colon. Butyrate, propionate, and acetate are SCFA metabolites that have gained interest in recent years due to their proposed health benefits. Butyrate is mainly utilized by the epithelial cells, whereas acetate and propionate can be transported across the epithelial barrier to become systemically available in low levels via the bloodstream as depicted in Figure 2 (106).

Upon absorption by the colonic epithelial cells, SCFA promote several functions of the epithelial barrier. The mucus layer covering the epithelial cells is essential to maintain epithelial barrier integrity. SCFA enhance the mucus secretion by upregulating the expression of mucin 2 (107). Acetate, produced in high levels by *Bifidobacterium* and *Bacteroides* species, increases the expression of genes related to mucus and support goblet cell differentiation (108–110). In addition, SCFA are known to protect against inflammatory insults and fortify the tight junction barrier (111). Promoting and enhancing the epithelial integrity may be of relevance in preventing allergic diseases, as a disrupted intestinal epithelial layer could lead to a compromised local tolerance response in which food allergens are able to reach underlying immune cells intact (112).

In addition, SCFA interact with DC and T cells and therefore modulate inflammatory immune responses. Many of the protective effects of SCFA have been attributed to the interaction with G protein-coupled receptors (GPR) present on intestinal epithelial cells and immune cells (113). Moreover, GPR-independent regulation of the immune response via T cell modulation has been shown in a murine model (114). In this model, SCFA regulate cytokine production via mammalian target of rapamycin (mTOR) by inhibiting histone deacetylase (HDAC) in T cells. In a previous study, butyrate effectively inhibited several HDACs in various cells, among which those that promote the transcription of FoxP3 in T cells, leading to increased expression of this hallmark transcription factor of regulatory T (T_reg_) cells (115, 116). In addition, inhibition of maturation and differentiation of macrophages and DCs has been demonstrated (117). Suppression of inflammatory responses by butyrate was shown to involve inhibition of the NF-κB pathway in inflammatory cells such as macrophages in the lamina propria (118).

Interestingly, recently it was found that the microbiome of infants who develop allergic diseases during childhood have a reduced genetic potential for butyrate production from complex carbohydrates, supporting the importance of SCFA production in protecting the infant from developing allergic diseases (119). Therefore, supporting the microbial development may be of interest in infants more susceptible to developing allergic diseases (120, 121). All together, as bacterial metabolites of HMOS, SCFA may contribute to the immunomodulatory and protective effects against allergic disease development.

## HMOS Strengthening the Intestinal Epithelial Integrity

Beyond their fermentation products, HMOS themselves may directly provide protection from intestinal epithelial barrier dysfunction (122), by promoting epithelial barrier maturation and mucus production (75) (illustrated in Figure 2). A mixture of human milk derived HMOS was shown to increase mucus production after 24 h of *in vitro* treatment in two different intestinal epithelial cell lines. The improved mucus production was linked to an upregulation of *Muc2*. In addition, apart from increased mucus production, HMOS could protect against pathogen induced barrier disruption as determined by means of transepithelial electrical resistance (TEER) (123). Furthermore, pollution induced loss of epithelial barrier integrity could be prevented by scGOS and 3'GL as measured in both TEER values and luciferase yellow flux across the intestinal epithelial monolayer in Caco-2 cells (124, 125). It was also demonstrated that supplementation with scGOS resulted in a significant increased rate of tight junction reassembly (124). Interestingly, the galactosyllactose with a β1–3 glycosidic linkage was effective in protecting the intestinal barrier function, whereas the galactosyllactose with an α1–3 glycosidic linkage did not prevent the deoxynivalenol (DON)-induced disrupted intestinal barrier (125). The protective effect of 3'GL on the intestinal epithelial barrier under challenge is structure-specific, which supports the notion that it is critical to understand the function and diversity of the structures within the total pool of HMOS, including the specific benefits of 3'GL within early life nutrition. These studies show that HMOS may directly promote proper development of the intestinal barrier, which strengthens the physical barrier between the intestinal epithelium and the gut content, contributing to lower antigenic load and mucosal homeostasis, which may help to decrease sensitization to food allergens.

In addition to this, the immunologic effects that are mediated through interaction between the intestinal epithelium and the underlying mucosal immune system should be addressed. Administration of synthetic HMOS 6'SL to antigen-antibody complex activated intestinal epithelial cells *in vitro* and resulted in a dose-dependent decrease of IL-8 and CCL20 secretion. Whereas, administration of 2'FL selectively reduced the secretion of CCL20 from the two cell lines used in this study (38). Similarly, a decrease of cytokine and chemokine production was observed upon TNFα stimulation of these cells after 6'SL exposure. Furthermore, comparable outcomes were observed for 3'GL, 4'GL, and 6'GL in an *in vitro* model for the infant intestinal epithelium (50). However, this decrease in cytokine production was not observed when two different intestinal cell lines were exposed to 2'FL (38). Additionally, it was observed that 3'SL, which is an isomer of 6'SL, downregulated the production of pro-inflammatory cytokines in Caco-2 intestinal cells by inhibition of the NF-κB pathway in a PPARγ dependent manner (126). These observations indicate that different functional groups and structures of HMOS exert the anti-inflammatory effects via different mechanisms. Silencing exaggerated or unwanted epithelial cell activation is essential for maintaining mucosal homeostasis.

Data indicated that mice, fed a diet supplemented with GOS for 2 weeks prior to exposure to DON, maintain their normal cellular distribution, as measured by villus height in the proximal small intestine (124). A study in suckling rats investigated the effects of 2'FL on mucosal immunomodulation (19). After treatment with 2'FL for 16 days an overall lower presence of inflammatory cytokines in the intestines compared to a reference group was observed, whereas the ratio of T_H_1/T_H_2 cytokines remained unchanged. In addition, the height and area under the villi present in the intestines was significantly increased upon supplementation with 2'FL, pointing to a positive effects of this prebiotic on intestinal growth (19). This is linked to the observation that 2'FL and scGOS/lcFOS in early life alter gut microbiome development while supporting vaccination responses (18, 127, 128).

In the light of NEC, especially sialylated oligosaccharides have shown promising outcomes *in vivo* in prevention and development of necrotic intestinal lesions (122). Several studies in neonatal rats have reported reduced pathology scores upon intervention with HMOS mixture (129), or single HMOS alone (130, 131). Although sialylated oligosaccharides have been identified as the protective agents (129), intervention with 2'FL has also resulted in a reduced pathology score in rats (130). Dietary supplementation of 2'FL in preterm pigs had no significant effects on intestinal structure, digestive function and the development of NEC (132). Nonetheless, pooled HMOS, rather than single HMOS, have consistently shown to be most effective in preventing development of NEC (122).

Moreover, it has been shown that HMOS provision early in life can protect against the development of autoimmune diabetes in NOD-mice (133). The number of *in vivo* studies looking into the immunomodulatory effects of single HMOS are rather limited, and currently restricted to only the simple short chain structures. In a murine model for hen's egg allergy, 2'FL or 6'SL were found to reduce allergy symptoms in association with the induction of IL-10^+^ T_reg_ cells (39). Prebiotic mixtures, such as scGOS and lcFOS, have been studied more extensively for immunomodulatory effects *in vivo*, showing promising results with regards to preventing allergic diseases, such allergic asthma and food allergy and these effects also link to the induction of T_reg_ responses (134–137). This implies a need for additional *in vivo* studies to gain insight in the properties of (single) HMOS to modulate gut maturation and the development of the mucosal immune system. Combining these studies, the direct effects of HMOS on the intestinal epithelial integrity and activation status and possibly the mucosal immune system are only started to be elucidated. The exact mechanisms and pathways involved are not yet fully understood. However, some of the receptors involved in HMOS signaling are identified and will be discussed in the following section.

## HMOS Bind to and Act as Receptors

One potential role of HMOS to modulate the infant's immune system is through receptor binding properties. In fact, multiple classes of human receptors have been described to interact with specific structures of HMOS, as summarized in Table 1. These receptors are mainly expressed by innate, adaptive immune cells and epithelial cells, they may therefore play a key role in mucosal immunomodulatory effects of HMOS (145).

**Table 1:** Overview of HMOS binding receptors, potentially involved in immunomodulation.

**HMOS identified as ligands**	**Receptor**	**Expression of receptor on**	**Function of receptor**	**References**
2'FL, 3FL, LNFP-III, LNFP-IV, LNDFH-I	DC-SIGN	Antigen presenting cells	Antigen presentation	(138–140)
3'SL and 6'SL	Siglec 5, 9	Neutrophils, monocytes, dendritic cells	Immune signaling	(138, 141)
LNnT, LNT, LNFP-II, LNFP-III, LNDFH	Galectin 1, 2, 3, 7, 8, 9	Intestinal cells, lymphocytes, antigen presenting cells	Immune signaling	(142–144)
2'FL and 3'SL	TLR4	Most cell types, mainly immune cells	Pathogen detection	(15, 16)

*Adapted from Triantis et al. (145)*.

### Glycan Receptors

Glycan-binding receptors, also known as lectins, are particularly effective in binding HMOS. Many of the receptors belonging to the lectin family are involved in modulation of immune pathways. Lectin receptors consist of several subcategories, such as: membrane bound C-type lectins, sialic acid binding immunoglobulin-like lectins (Siglecs) and soluble type galectins.

The C-type lectin receptor dendritic cell-specific ICAM-grabbing non-integrin (DC-SIGN) is present on the surface of DCs and macrophages. It is usually involved in phagocytosis of pathogens upon recognizing pathogen-related glycoproteins. DC-SIGN has an affinity for HMOS containing α-linked fucose residues (138). A high affinity for 2'FL and 3FL (2 major structures of HMOS) may be of distinct physiological relevance in modulating immune responses in infants. DC-SIGN is expressed by cells in the gastrointestinal tract (139) and this receptor can promote allergen uptake by DCs. This may lead to subsequent T_H_2 cell polarization as seen in patients with atopic dermatitis (146). Therefore, even though DC-SIGN can confer protective regulatory immunity in a pre-clinical model for auto-immune disease (147), DC-SIGN signaling may be involved in the sensitization phase of allergic diseases as allergens are capable of DC activation via DC-SIGN binding (148). An HMOS mixture derived from human milk was found to lower the expression of DC-SIGN on DC (140). This indicates that HMOS may be able to reduce DC-SIGN driven allergic sensitization through suppression of DC-SIGN expression on DC and via blocking the DC-SIGN receptor.

Siglecs are expressed by several immune cells that are involved in allergic effector responses, such as eosinophils and mast cells. Siglecs have been associated with binding of sialylated HMOS, although previous results show only affinity of siglec-1, -5, -7, -9, and -10 to 3'SL and 6'SL (141), and more recent data show a more limited binding affinity of Siglecs for HMOS (138). Siglec-9 provides low binding affinity for 3'SL and 6'SL, while siglec-5 has very low affinity for only 3'SL. This study found no other Siglecs to bind sialylated HMOS (138). Hence, the presence of sialic acid alone is not sufficient to ensure functional binding to a Siglec receptor (138). Siglec-7 and siglec-8 have been associated with allergy related immune mechanism (149, 150) making these potential targets for immune modulation by HMOS in relation to allergy prevention.

Galectins are another group of β-galactoside-binding receptors that bind carbohydrate moieties or glycan structures present on proteins. Moreover, galectins are expressed on and/or secreted by several immune cells and intestinal epithelial cells (151). These receptors can directly forward signals into the cell upon binding to a ligand, but galectins can also be secreted from cells (152). In the secreted form, galectins can act as ligands and bind to receptors, such as TIM-3 and CD44 on other mucosal immune cells (153). Galectins such as galectin-9 have shown to induce T_reg_ cells (154–156). The binding of HMOS to galectins may directly modify galectin release and affect interactions of galectins with other cells, potentially resulting in immune modulation. Of the thirty-two different HMOS structures tested for binding to four galectins (galectin-1, -3, -7, and -9) (142), a total of 25 of these structures were recognized by all four galectins. Significant differences in affinity for each HMOS were observed, i.e., 2'FL, 3'SL, and LNnT were shown to bind galectins, whereas 3FL and 6'SL did not. 2'FL, the most common HMOS in human milk, binds with moderate-to-high affinity to all four galectins, while 3FL a structure very similar to 2'FL, not or weakly binds to any of the four galectins included in the study (142). Similar results were obtained in a different report, including galectin-1, -3, and -7 (143). These findings are supported by a previous study (144), suggesting that all included galectins showed affinity for LNnT, but had no affinity for 6'SL. This study also highlighted the evolutionary conserved binding affinity of galectins for glycans. Galectin-9 is a particularly promising target in allergy prevention strategies, as exposure of intestinal epithelial cells to scGOS/lcFOS together with bacterial CpG DNA or synthetic CpG ODN promoted the secretion of galectin-9 *in vitro*, which resulted in enhanced secretion of IFNγ and IL-10 production by underlying immune cells (154, 157). These cytokines are related to a regulatory type of T_H_1 polarization and suppress T_H_2 cell activation. Experiments with dietary interventions including scGOS/lcFOS enhanced local and/or systemic galectin-9 levels in murine and human allergy in association with symptom reduction (137). Furthermore, galectins can become systemically available and dampen allergic effector responses as shown in a murine model of food allergy (137).

### Pattern Recognition Receptors

Toll-like receptors (TLR) are a family of receptors known to sense common molecules of pathogenic or commensal microorganisms, such as TLR4 ligand lipopolysaccharide (LPS) or TLR9 ligand bacterial CpG DNA. Decreased formation of the three-component complex TLR4, CD14, and LPS, inhibits subsequent pro-inflammatory immune signaling (158). Xiao et al. showed an increase in LPS receptor TLR4 mRNA expression upon stimulation with pooled HMOS isolated from human milk in monocytic derived dendritic cells (moDC) *in vitro*, yet protein levels of this receptor were not increased (140). In addition to affecting TLR4 transcription, HMOS suppress the expression of cluster of differentiation (CD)14, a coreceptor of TLR which is necessary to recognize LPS. 2'FL significantly suppresses CD14 in intestinal epithelial cells (16). In contrast to suppression of inflammation via TLR4 by 2'FL, pro-inflammatory properties related to TLR4 modulation have been described for synthetic 3'SL. In a TLR4-dependent manner, 3'SL was shown to induce intestinal inflammation (15). This pro-inflammatory effect of 3'SL can be explained by mimicking possible structural aspects of pathogenic bacteria, thereby educating and preparing the immune system for possible pathogenic encounters later in life. However, the phenotypical changes of DCs by 3'SL may have been due to LPS contamination of the oligosaccharide during synthesis, since pre-exposure to LPS may contribute to TLR4 silencing (159). However, LPS-containing bacteria are normal components of a healthy intestinal microbiome (160). In this respect, the low level of endotoxins present in purified HMOS used in *in vivo* studies would be minimal compared to the vast amount of endotoxin triggers the infant receives directly after birth. The contradicting results regarding HMOS-induced modulation of TLR4 show that we are only beginning to elucidate the possible immunomodulatory effects of HMOS. In addition, as synthetic (s)HMOS are either derived from enzymatically-processed lactose or produced by *E. coli*. In the latter situation a second possible immune trigger from bacterial byproducts may add to the biological effects of sHMOS structure. The origin of HMOS may influence the immunomodulatory effect, therefore an overview of the source and main outcomes of the studies referred to in this review is provided in Table 2.

**Table 2:** Overview of studies included in this review, which describe effects of non-digestible oligosaccharides (NDO) on immune function.

**References**	**Model**	**NDO**	**Main effect of intervention**
***In vitro***			
Gnoth et al. (42)	Caco-2 cells	Isolated HMOS	Neutral HMOS are transported across intestinal epithelia via receptor-mediated transcytosis as well as by paracellular flux, while acidic HMOS are translocated solely via paracellular pathways
Eiwegger et al. (161)	cord blood T cells	Isolated HMOS	Acidic HMOS increased the percentage of IFN? and IL-13 producing T cells as well as CD25+ T cells. IgE and IgG1 production was unaffected
Coppa et al. (101)	Caco-2 cells	Isolated HMOS	Acidic HMOS showed anti-adhesive effects on all 3 intestinal pathogens. Neutral HMOS showed anti-adhesive effects on 2 out of 3 tested pathogens
He et al. (49)	Fetal small intestinal samples	Isolated HMOS	HMOS from colostrum samples were able to attenuate mucosal response to surface inflammatory stimuli, and enhanced maturation of intestinal mucosa
Xiao et al. (140)	human moDCs	Isolated HMOS	HMOs limited LPS maturation of moDCs. HMOS-conditioned moDCs promoted T_reg_ generation
Newburg et al. (50)	T84 cells, H4 cells, NCM-460	Isolated HMOS and GOS	HMOS attenuated surface inflammatory stimuli. HMOS and GOS attenuated NF-κB signaling
Eiwegger et al. (162)	Caco-2 cells	Isolated HMOS and scGOS + lcFOS and AOS	Acidic HMOS increased IFN? and IL-10 secretion and suppressed T_H_2 cytokine production in T cells from peanut allergic patients
He et al. (16)	T84 cells, H4 cells	Isolated HMOS, 2'FL^3^, LNFP-I^3^, 3'SL^3^ and 6'SL^3^	HMOS and 2'FL inhibited LPS-TLR4 signaling via suppressed CD14 expression. No significant results for any of the other tested NDOs
Holscher et al. (75)	Caco-2Bbe cells, HT-29 cells	Isolated HMOS, 2'FL^1^, 3'SL^2^ and 6'SL^1^	Single HMOS and isolated HMOS decreased proliferation in pre-confluent cells, but increased cell differentiation. isolated HMOS decreased apoptosis and necrosis
Akbari et al. (124)	Caco-2 cells	GOS	GOS improved tight junction assembly and DON induced loss of transepithelial resistance was prevented
De Kivit et al. (154)	T84 cells, HT-29 cells	scGOS + lcFOS	scGOS + lcFOS in combination with *B. breve* M-16V increased epithelial expression and secretion of galectin-9, and enhanced T_H_1 and T_reg_ polarization
Hayen et al. (157)	HT-29 cells	scGOS + lcFOS and scFOS + lcFOS	Both mixtures induced enhanced IFN? and IL-10, but suppressed IL-13 and TNFα secretion. scFOS + lcFOS enhanced T_H_1 and T_reg_ response in a peanut-specific co-culture (HT-29/PBMC) model
Zenhom et al. (126)	Caco-2 cells	FOS and 3'SL^3^	Both decreased levels of inflammation, as IL-12 secretion and mRNA expression of IL-12p35, IL-8, and TNFα was reduced in a dose- and time-dependent manner
Perdijk et al. (163)	human moDCs	GOS, 2'FL^1^ and 6'SL^1^	None of the oligosaccharides influenced DC differentiation and LPS-induced maturation
Yu et al. (17)	Hep-2 cells, HT-29 cells	2'FL^2^	2'FL attenuated *C. jejuni* invasion in both cell lines
Perdijk et al. (159)	human moDCs	3'SL^1^	3'SL mediated NF-κB activation via TLR4 induction was explained by LPS contamination
Zehra et al. (38)	T84 cells, HT-29 cells	2'FL^2^ and 6'SL^2^	2'FL inhibited CCL20 secretion from epithelium upon antigen-antibody complex stimulation. 6'SL inhibited IL-8 and CCL20 secretion from epithelium upon antigen-antibody complex stimulation
Holscher et al. (74)	Caco-2Bbe cells, HT-29 cells	LNnT^3^, 2'FL^3^ and 6'SL^3^	All HMOS inhibited cell proliferation in undifferentiated cell cultures. 2'FL increased alkaline phosphatase and sucrase activity. LNnT increased transepithelial resistance
Varasteh et al. (125)	Caco-2 cells	3'GL^3^, 4'GL^3^ and 6'GL^3^	3'GL prevented loss of transepithelial resistance upon DON exposure, 4'GL and 6'GL had no effect
**Pre-clinical**			
Xiao et al. (133)	Mice	Isolated HMOS	HMOS intervention delayed and suppressed type 1 diabetes development and reduced development of severe pancreatic insulitis in NOD-mice
Wu et al. (123)	Mice	Isolated HMOS	HMOS increased mucin expression, whereas intestinal permeability was decreased
Jantscher-Krenn et al. (129)	Mice	Isolated HMOS and GOS	HMOS reduced NEC pathology scores, the effects were attributed to DSLNT in the HMOS mixture
Yu et al. (131)	Rats	Isolated HMOS, GOS and synthetic disialylated-GOS	HMOS and sialylated-GOS reduced NEC pathology scores. GOS had no effect on NEC development
Autran et al. (130)	Rats	Isolated HMOS, GOS and synthetic disialylated-GOS	HMOS and sialylated-GOS reduced NEC pathology scores. GOS had no effect on NEC development
Comstock et al. (164)	Pigs	Isolated HMOS, 2'FL^3^, 3FL^3^, 3'SL^3^, 6'SL^3^, LNFP-III^3^ and LNnT^3^	HMOS stimulation IL-10 production by PBMCs. Fucosylated HMOS decreased proliferation of HMOS. Sialylated HMOS increased PBMC proliferation, although less CD4+ cells were observed
Akbari et al. (124)	Mice	GOS	GOS treatment stabilized villus height upon DON exposure
Verheijden et al. (30)	Mice	GOS	GOS prevented induction of airway eosinophilia and T_H_2 related cytokine concentrations in lung, similar to budesonide treatment in house-dust mite allergy
Verheijden et al. (135)	Mice	GOS	GOS decreased IL-33 secretion and expression in HDM-induced asthma
Verheijden et al. (165)	Mice	GOS	GOS decreased CCL5 and IL-13 concentration in lung tissue from HDM-induced allergic asthma mice, similar to budesonide treatment
Djouzi and Andlueux (23)	Rats	GOS and FOS	GOS and FOS decreased pH in caecum, increased total SCFA concentration
Verheijden et al. (31)	Mice	scFOS + lcFOS	scFOS + lcFOS in combination with *B. breve* M-16V prevented house-dust mite induced airway inflammation
De Kivit et al. (137)	Mice	scGOS + lcFOS	scGOS + lcFOS in combination with *B. breve* M-16V induced reduced acute allergic skin response, and higher concentrations of galectin-9, which was associated with allergy prevention
De Kivit et al. (166)	Mice	scGOS + lcFOS	scGOS + lcFOS in combination with *B. breve* M-16V in an ovalbumin allergic mouse model, reduced allergic symptoms and increased galectin-9 serum levels. DC activation and T_H_2 frequency were normalized in allergic mice
Schouten et al. (134)	Mice	scGOS + lcFOS + AOS	Prebiotic mixtures enhanced percentages of T_H_1 cells and decreased Th2 cell percentages were observed. Strong reduction in allergic skin reaction. CD25+ T_reg_ cells were involved in the tolerance induction effect
Kerperien et al. (29)	Mice	scGOS + lcFOS and AOS	Only NDO mixtures reduced allergic skin response, whey-IgG1 levels, T_H_2 and T_H_17 mRNA expression, and increased Foxp3+ cells
Kerperien et al. (136)	Mice	scGOS + lcFOS + AOS	Prebiotic mixtures increased mRNA expression of IL10, TGFβ and Foxp3, and acute allergic skin response was 50% lower in whey allergic mice when fed the prebiotic mixture. These protective effect were depended on IL10 and TGFβ
Xiao et al. (127)	Mice	scGOS + lcFOS + 2'FL^2^	NDOs enhanced influenza vaccine response, higher levels of IgG1, IgG2a, and activated B cells were observed
van den Elsen et al. (128)	Mice	scGOS + lcFOS + 2'FL^2^	NDOs improved vaccine-specific antibody response and modulated gut microbiota composition
Yu et al. (17)	Mice	2'FL^2^	2'FL attenuated *C. jejuni* colonization, weight loss and inflammatory cytokines
Cilieborg et al. (132)	Pigs	2'FL^3^	2'FL intervention did not result in observed differences in bacterial colonization, intestinal function and NEC pathology
Xiao et al. (18)	Mice	2'FL^2^	2'FL improved humoral and cellular immune response to influenza vaccination
Azagra-Boronat et al. (19)	Rats	2'FL^3^	2'FL increased plasma IgE and IgA levels. Increased intestinal villus height. Higher *Lactobacillus proportion* in cecum
Weiss and Hennet (103)	Mice	3'SL^3^	3'SL induced higher degree of resistance to dextran sulfate sodium-induced colitis
Kurakevich et al. (15)	Mice	3'SL^3^	3'SL increased colitis, via TLR4 signaling
Castillo-Courtade et al. (39)	Mice	2'FL^2^ and 6'SL^2^	2'FL and 6'SL attenuated ovalbumin induced allergic symptoms like diarrhea, hypothermia, mast cell number in the intestine, and increased induction of IL-10 producing T_reg_ cells
**Clinical**			
Newburg et al. (32)	Infants	HMOS in human milk	Higher 2'FL and LNF-I to 3FL and LNF-II ratios in human milk correlated with more protection against diarrhea in infants
Sjögren et al. (35)	Infants	HMOS in human milk	Neutral HMOS concentration in human milk is not related to maternal allergy status nor allergy development in children
Bode et al. (33)	Infants	HMOS in human milk	Higher concentrations of HMOS in human milk were correlated to decreased risk of HIV transmission from mother to child. However, higher concentrations of 3'SL were found in HIV transmitting woman
Wang et al. (88)	Infants	HMOS in human milk	Breastfed infants had relative higher abundances of Bacteroides, and lower proportions of *Clostridium, Streptococcus, Enterococcus* and *Veillonella* than infants fed formula milk
Kuhn et al. (34)	Infants	HMOS in human milk	Higher concentrations of 2'FL and LNF-I were found in human milk from HIV non-transmitting woman
Sprenger et al. (36)	Infants	HMOS in human milk	FUT-2 associated oligosaccharides in human milk in infants at high risk of allergy development, and born via C-section are associated with lower risk of IgE-associated eczema
Seppo et al. (37)	Infants	HMOS in human milk	Low LNFP-III concentrations in human milk was related to an increased likelihood to develop cow's milk allergy, compared high concentrations of LNFP-III in infants
Grüber et al. (44)	Infants	Neutral oligosaccharides + AOS	Prebiotic supplemented formula resulted in a significant lower rate of atopic dermatitis compared normal formula in infants. Incidence of atopic dermatitis in prebiotic supplemented infants was in a similar range compared to breast fed infants
Moro et al. (27)	Infants	GOS and FOS	GOS and FOS dose-dependently increased in Bifidobacteria and Lactobacilli, in infants receiving prebiotic supplemented formula compared to non-supplemented formula
Arslanoglu et al. (28)	Infants	scGOS + lcFOS	Infants receiving scGOS + lcFOS had a lower incidence of allergic manifestations, in addition, fewer physician-diagnosed respiratory tract infections, fever episodes, and antibiotic prescriptions were recorded
De Kivit et al. (137)	Infants	scGOS + lcFOS	scGOS + lcFOS in combination with *B. breve* M-16V induced higher serum galectin-9 levels, which is associated with allergy prevention
Goehring et al. (167)	Infants	GOS + 2'FL^3^	GOS + 2'FL supplemented formula fed infants had similar plasma inflammatory cytokine concentrations compared to breast fed infants. Infants fed with the GOS diet had significantly increased levels of inflammatory cytokines present in plasma

*As HMOS has different origin which may influence the immunological outcome, when possible the origin of the used HMOS was noted. Biological isolated HMOS^1^, chemically synthesized^2^, bacterial fermentation/synthesis^3^ or source unknown. Studies are sorted based on model subgroup (e.g., in vivo), NDO and year of publication*.

### Pathogen Binding

Besides binding to receptors on the cell membrane, HMOS can act as soluble receptors and bind to several pathogenic bacteria, thereby preventing binding to the intestinal epithelium and subsequent infection (101). Both *in vitro* and *in vivo* studies show that 2'FL attenuated *Campylobacter jejuni* infection (17, 168). However, Coppa et al. did not find inhibition of adhesion of *Escherichia coli, Vibrio cholerae* and *Salmonella fyris* in an *in vitro* intestinal epithelial setting with 2'FL (101). Nonetheless, inhibition of adhesion was observed with 3'SL, 6'SL and 3FL and combinations of these sHMOS. There was a diminished growth of *Streptococcus agalactiae* (group B *Streptococcus*) upon incubation with human pooled natural HMOS, that was attributed to the neutral fraction of the HMOS (169). This effect was supported by other studies, as pooled HMOS inhibited growth of group B *Streptococcus* (GBS) and prevented biofilm formation, although the effects of single HMOS were GBS strain specific (170–172). In this study, the effects of HMOS were compared to scGOS. scGOS did not diminish the growth of group B *Streptococcus* (169), showing that the structures in scGOS in this respect do not exert similar effects as the mentioned HMOS subtypes. These studies indicate that HMOS can also function as decoy receptors, thereby inhibiting growth and adhesion of pathogens in the gastrointestinal tract.

As antibiotic resistance is a growing problem, alternative antibacterial treatments are being investigated (173), including the use of HMOS to potentiate antibiotic functioning (174). It has been recently demonstrated that when exposed to HMOS, GBS becomes sensitive for trimethoprim, an antibiotic to which these bacteria are normally resistant. A significant decrease in metabolic pathways related to membrane construction was observed (175). Furthermore, HMOS were able to sensitize GBS to several antibiotics, such as erythromycin, gentamycin and clindamycin. In addition, an increased sensitivity to gentamycin, when combined with HMOS, in *Staphylococcus aureus* and *Acinetobacter baumanii* was also observed. However, these potentiating effects were obtained for β-lactams and glycopeptides (176). Next to the above reported antibacterial properties, similarly some viral inhibiting interactions have been described (177). These interactions include binding of 2'FL to conserved epitopes, which are involved in binding to host cells, on norovirus (178, 179). Next to 2'FL, also 3'SL and 6'SL showed to inhibit cell binding in a rotavirus *in vitro* model (180). Some promising results of HMOS intervention have even been observed for influenza and HIV infections (177).

## HMOS Interact with Immune Cells

HMOS have been detected in the blood, feces and urine of breastfed term and preterm infants (181–184). In breastfed infants, HMOS concentrations in urine appear to be around 10 times higher than in serum (184), which can be explained by clearance of substances from a larger volume of blood and accumulation in a small volume of urine. Direct effects have been demonstrated *in vitro* in bone marrow-derived dendritic cells (BMDC) treated with 2'FL. There was an increase in the percentage of CD40+ and CD86+ BMDCs upon exposure to 2'FL (18). Direct modulation of human moDCs was not found for 2'FL, 6'SL and scGOS (163), but the idea of possible moDC modulation via other HMOS cannot be excluded. BMDC exposed to 2'FL and stimulated by influenza vaccination had a greater capacity to induce CD4+ T cell proliferation in fresh whole splenocytes (18). Low concentrations of a mixture of acidic HMOS, purified from human milk, can alter cytokine production in cord blood mononuclear cells (CBMC) (161). The production of IFNγ and IL-10 in CBMCs was increased upon exposure to acidic HMOS, while IL-13 production remained unaltered, pointing to skewing of the balance toward a regulatory type T_H_1 response. Similar effects were observed in a prior study exposing CBMC to acidic HMOS, which resulted in decreased IL-13 production in T cells (162). Mast cell function and direct effects of HMOS on mast cell degranulation were investigated in a murine food allergy model (39). *In vitro* exposure of bone marrow-derived mast cells to 6'SL resulted in significant inhibition of IgE-dependent mast cell degranulation, but only at a relatively high concentration of 1 mg/mL. However, in this same study, 2'FL did not significantly inhibit mast cell activation. Both 6'SL and 2'FL induce IL10^+^ T_reg_ cells and thereby indirectly stabilize the degranulation of mast cells, in association with reduced food allergy symptoms (39). Hence, HMOS may have the capacity to modulate the immune response via various mechanisms, as indicated by the direct effects of HMOS on several immune cell types.

In the above described murine model for food allergy, 2'FL and 6'SL reduced food allergy symptoms via inducing T_reg_ cells and modulating mast cells (39). After 2'FL and 6'SL treatment during challenge in ovalbumin sensitized mice enhanced the capacity of CD4+CD25+ T_reg_ cells to inhibit mast cell degranulation *ex vivo* (39), indicating that specific sHMOS support T_reg_ cell function. Similar results were found using scGOS and lcFOS in combination with acidic oligosaccharides or *B. breve* in prevention of food- (29, 166) or asthma-allergy in mice (31, 165). In piglets, either sow-reared or formula fed, peripheral blood mononuclear cells (PBMCs) were isolated (164). PBMCs from formula fed piglets showed more proliferation than sow-reared piglets upon LPS stimulation *ex vivo*, while *ex vivo* addition of sHMOS 2'FL normalized this increased proliferation. The percentage of T helper cells was higher in formula fed piglets compared to sow-reared piglets. *Ex vivo* added synthetic fucosylated and sialylated oligosaccharides downsized the expansion of the T_H_ cell population in the formula fed piglets, while the cytotoxic T cell population remained unaffected by *ex vivo* sHMOS treatment (164). These results indicate that fucosylated and sialylated oligosaccharides may possess immune regulatory properties, potentially modulating an allergic inflammatory response.

Although clinical trials in this area of research are scarce, data from an initial study indicate that addition of 2'FL to infant formula lowers concentrations of pro-inflammatory cytokines in plasma compared to infants fed a control formula (167). In addition, the decrease of these cytokines in the 2'FL supplemented infants was comparable to the low level of inflammatory cytokines that was measured in plasma of breastfed infants (167). As such, it should be carefully considered whether the effects observed in any of the *in vivo* and clinical studies are caused by a direct effect of the HMOS or indirect immunomodulatory effects as a result of microbiome modulation.

A convincing body of evidence is missing to ascribe clear immune development properties to HMOS and individual HMOS structures, since only a small number of *in vivo* studies describe immunomodulatory properties and immune maturation. In addition, the exact properties of the different groups of HMOS to modulate the immune system are not clear. Therefore, several studies illustrating immunomodulatory effects of scGOS and lcFOS have been described here and summarized in Table 2, as they may propose a framework in which future research could focus to elucidate immune related mechanisms affected by HMOS. As synthetically produced HMOS have become available recently, studying these may contribute to acquiring knowledge of the exact properties of HMOS and their specific functional groups in more detail and promote research focussing on allergy prevention. Development of adequate *in vitro* models for allergic sensitization including intestinal epithelial cells and/or dendritic cells, may help understanding the direct immunomodulatory effects of HMOS and their possible role in allergy prevention.

## Conclusion

The increasing prevalence of allergic diseases has sparked interest in the role of early life nutrition and allergy development. Dietary components drive early life microbiome development as well as gut and immune maturation. HMOS in breast milk exhibit various microbiome modulating as well as mucosal immune maturation properties, which are not yet fully understood. However, in recent years several pathways involved in the effects of HMOS have been elucidated, including their capacities to fortify the microbiome composition and the release of fermentation products including SCFAs, as well as direct binding to pathogens and interactions with the gastrointestinal epithelium and local and systemic immune cells (as illustrated in Figure 2). Specific structural groups of HMOS may target several aspects of the immune system and modify immune function, thereby highlighting the need for further research on this topic. In addition, a more diverse mixture of oligosaccharide structures in neonatal formula nutrition may more closely resemble the HMOS composition as available in human breast milk and provide extra benefit for the child. Future research should focus on uncovering the mechanisms and pathways by which HMOS and the specific functional groups present in these HMOS may exert immunomodulatory actions. Ultimately, it would be of utmost value to identify whether specific HMOS structures are capable of contributing to early life allergy prevention.

## Author Contributions

MZ and NW have written the review. JG, GF, BL, and LW supervised the program. BL and LW have discussed and edited the manuscript. BS made specific contribution to the program with regard to functional oligosaccharides. All authors listed have approved for publication.

## Conflict of Interest

JG is head of the Division of Pharmacology, Utrecht Institute for Pharmaceutical Sciences, Faculty of Science at Utrecht University and partly employed by Danone Nutricia Research B.V. BS and BL are employed by Danone Nutricia Research B.V. BS has an associated position at Utrecht Institute for Pharmaceutical Sciences, CBDD, Faculty of Science at Utrecht University. BL is affiliated at and leading a strategic alliance between Danone Nutricia Research B.V. and the University Medical Centre Utrecht/Wilhelmina Children's Hospital. The remaining authors declare that the research was conducted in the absence of any commercial or financial relationships that could be construed as a potential conflict of interest.
